# Nanotechnology Integration for SARS-CoV-2 Diagnosis and Treatment: An Approach to Preventing Pandemic

**DOI:** 10.3390/nano11071841

**Published:** 2021-07-16

**Authors:** Syed Mohammed Basheeruddin Asdaq, Abu Md Ashif Ikbal, Ram Kumar Sahu, Bedanta Bhattacharjee, Tirna Paul, Bhargab Deka, Santosh Fattepur, Retno Widyowati, Joshi Vijaya, Mohammed Al mohaini, Abdulkhaliq J. Alsalman, Mohd. Imran, Sreeharsha Nagaraja, Anroop B. Nair, Mahesh Attimarad, Katharigatta N. Venugopala

**Affiliations:** 1 Department of Pharmacy Practice, College of Pharmacy, AlMaarefa University, Dariyah, Riyadh 13713, Saudi Arabia; 2 Department of Pharmacy, Tripura University (A Central University), Suryamaninagar 799022, Tripura (W), India; abumd97@gmail.com; 3 Department of Pharmaceutical Science, Faculty of Pharmacy, Universitas Airlangga, Surabaya 60115, Indonesia; rr-retno-w@ff.unair.ac.id; 4 Department of Pharmaceutical Science, Assam University (A Central University), Silchar 788011, Assam, India; 5 Department of Pharmaceutical Sciences, Faculty of Science and Engineering, Dibrugarh University, Dibrugarh 786004, Assam, India; bedanta1994@gmail.com (B.B.); tirnapaul97@gmail.com (T.P.); bhargavdeka98@gmail.com (B.D.); 6 School of Pharmacy, Management and Science University, Seksyen 13, Shah Alam 40100, Selangor, Malaysia; 7 Department of Pharmaceutics, Government College of Pharmacy, Bangalore 560027, Karnataka, India; vijay.joshi67@gmail.com; 8 Basic Sciences Department, College of Applied Medical Sciences, King Saud bin Abdulaziz University for Health Sciences, Alahsa 31982, Saudi Arabia; Maam670@gmail.com; 9 King Abdullah International Medical Research Center, Alahsa 31982, Saudi Arabia; 10 Department of Clinical Pharmacy, Faculty of Pharmacy, Northern Border University, Rafha 91911, Saudi Arabia; kaliqs@gmail.com; 11 Department of Pharmaceutical Chemistry, Faculty of Pharmacy, Northern Border University, Rafha 91911, Saudi Arabia; imran.pchem@gmail.com; 12 Department of Pharmaceutical Sciences, College of Clinical Pharmacy, King Faisal University, Al-Hofuf, Al-Ahsa 31982, Saudi Arabia; sharsha@kfu.edu.sa (S.N.); anair@kfu.edu.sa (A.B.N.); mattimarad@kfu.edu.sa (M.A.); kvenugopala@kfu.edu.sa (K.N.V.); 13 Department of Pharmaceutics, Vidya Siri College of Pharmacy, Off Sarjapura Road, Bangalore 560035, India; 14 Department of Biotechnology and Food Technology, Durban University of Technology, Durban 4001, South Africa

**Keywords:** COVID-19, nanotechnology, vaccines, sanitizers, biosensors

## Abstract

The SARS-CoV-2 outbreak is the COVID-19 disease, which has caused massive health devastation, prompting the World Health Organization to declare a worldwide health emergency. The corona virus infected millions of people worldwide, and many died as a result of a lack of particular medications. The current emergency necessitates extensive therapy in order to stop the spread of the coronavirus. There are various vaccinations available, but no validated COVID-19 treatments. Since its outbreak, many therapeutics have been tested, including the use of repurposed medications, nucleoside inhibitors, protease inhibitors, broad spectrum antivirals, convalescence plasma therapies, immune-modulators, and monoclonal antibodies. However, these approaches have not yielded any outcomes and are mostly used to alleviate symptoms associated with potentially fatal adverse drug reactions. Nanoparticles, on the other hand, may prove to be an effective treatment for COVID-19. They can be designed to boost the efficacy of currently available antiviral medications or to trigger a rapid immune response against COVID-19. In the last decade, there has been significant progress in nanotechnology. This review focuses on the virus’s basic structure, pathogenesis, and current treatment options for COVID-19. This study addresses nanotechnology and its applications in diagnosis, prevention, treatment, and targeted vaccine delivery, laying the groundwork for a successful pandemic fight.

## 1. Introduction

The severe acute respiratory syndrome coronavirus 2 (SARS-CoV-2) was responsible for the recent coronavirus 2019 outbreak (COVID-19) [1]. This pandemic has resulted in tense situations around the country, resulting in regulations such as social distance, lockdowns, and other measures. According to a report published in November 2002 in Guangdong, China, the first disease confronted by the world in the twenty-first century was SARS [2]. Other four epidemics, such as SARS, Ebola, Swine Flu, and Middle East Respiratory Syndrome, do not appear to be able to match the fatality produced by COVID-19 [2]. COVID-19 has infected around 189 million individuals worldwide, including over 4 million deaths (https://www.worldometers.info/coronavirus, retrieved on 15 July 2021). This COVID-19 has quickly turned an epidemic into a pandemic. SARS-CoV-2 is a spherical encapsulated virus with a diameter of 60–140 nm that contains positive-sense single-stranded RNA [(+) ssRNA] [3]. COVID-19 is made up of several components, including an extracellular (E) protein, a nucleocapsid (N) protein, a spike (S) glycoprotein, and a matrix (M) protein [4]. Corona virus’s globular protein has a size range of 70–90 mm [5]. S1 and S2 are two functional components of the S protein.

The [(+) ssRNA] interacts with N-protein to produce a helical nucleocapsid [6]. Fever, dry cough, loss of taste and smell, weariness, and difficulty breathing are some of the usual symptoms of COVID-19 infection. Ground-glass opacity in the lungs, pneumonia and other severe symptoms are examples. Some patients affected by COVID-19 are asymptomatic [7,8]. The reverse transcription-polymerase chain reaction (RT-PCR) technology is used to confirm the infection by detecting the COVID-19 virus’s genetic material in blood and discharge samples. Rapid Antigen test (RAT), or rapid antigen test, is another method of diagnosis [9]. The reverse transcription-polymerase chain reaction (RT-PCR) approach is regarded the superior test for COVID-19 due to its excellent accuracy and specificity [9]. Chest-computed tomography (Chest-CT) scans may be used by some professionals for diagnosis [9]. When an infected person sneezes, talks, or coughs, tiny particles or droplets known as aerosols are released into the environment through their mouth or nose, allowing the virus to spread. Anyone within 6–8 feet of that person can breathe it into their lungs, and it is a direct transmission mode [10]. Indirect contact manifests itself by contacting a surface where a patient may have sneezed or coughed [11]. SARS-CoV-2 has yet to receive a precise treatment. Asymptomatic or mildly symptomatic people, on the other hand, should isolate themselves in a well-ventilated room. They should use a medical mask and discard it after around 8 h. Individuals should get enough rest and drink enough drinks to be hydrated. Daily temperature and oxygen levels should be checked. Take medical help if any deterioration of symptoms is noticed. Sanitization of the individual and the room should be done frequently. When fever, weakness is examined, paracetamol along with Azithromycin and multivitamins are given. Employment of nanotechnology can be seen in the extremity of viral diseases such as COVID-19 [12]. Food and Drug Administration (FDA) gives voice to nanotechnology systems that provide different biological and physicochemical properties, compared to micro and macro stuff [12]. For instance, nanocarriers encapsulated drugs can control the release rate to the targeted sites, which lessens the toxicity and enhances biocompatibility in healthy tissues [13,14]. Nanomaterials help avoid viral spoliation by air and may help sterilize purposes even in a hospital environment. One of the most attention-seeking nanomaterials is gold nano-particles (i.e., AuNPs). Their quantum mechanical properties can be traversed by various forms of biosensors, mainly those that depend on electrochemical, plasmonic, and colorimetric estimation [15,16]. Nanotechnology-based therapy techniques provide a potential way to address constraints in COVID-19 diagnosis, and treatment [17]. Nanomaterials can also be employed in diagnostic to produce simple, quick, and low-cost approaches for detecting SARS-CoV-2. Nanosystems are used to deliver small biomolecules and antiviral to the pulmonary system in a regulated manner, like, to suppress viral spreading [17]. We will discuss the virus’s genetic morphological structure, etiopathogenesis, and current therapeutic intervention in this review. We also described and explored how nanotechnology could be used to fight this condition, as well as the most promising nanomaterials. We also expect that the nanotechnology-based strategies outlined in this study will aid researchers in developing novel COVID-19 diagnostic and treatment approaches.

## 2. Genetic Morphological Structure of SARS-CoV-2

**Figure 1:**
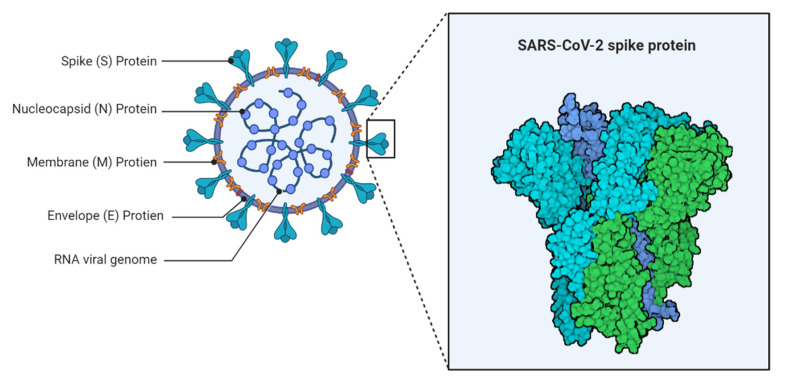
Structure of SARS-CoV-2.

SARS-CoV-2 is a crown-shaped, single-stranded RNA virus with active-sense single-stranded RNA. It belongs to the Coronaviridae family and has a diameter range of 600–1400 Aº [18]. An electron microscope can be used to observe extracellular and free particles [19]. The spike (S), envelope (E), membrane (M), and nucleocapsid (N) components of the virus are responsible for pathogenesis (Figure 1) [20].

By creating a nucleocapsid, the N protein aids in the replication process in the host cell. The three transmembrane segments of the M protein are crucial for the virus’s shape, size, and replication. The E protein plays a vital part in the virus’s pathogenic processes and the various stages of replication in infected cells [21]. In connection with this the S protein is made up of two subunits: S1 and S2. The subunits S1 play chief role for receptor binding and cell recognition, while the subunit S2 allows virus-cell fusion [22]. The structural analysis is required for the development of diagnostic procedures and vaccines [23].

## 3. Etiopathogenesis of SARS-CoV-2

The pathogenesis of the COVID-19 virus was disseminated to the host body by attachment, penetration, biosynthesis, maturity, and release of virus are all steps in the process [24]. The viral attachment with the interaction of viral spike protein and angiotensin-converting enzyme 2 (ACE2) receptor of the host cell. Direct membrane attachment or fusion occurs with the host receptor by undergoing proteolytic cleavage at the S1/S2 boundary, known to be penetration [25,26]. Then, in the host cell cytoplasm, the RNA is released, then RNA moves towards the nucleus and inside the nucleus; biosynthesis occurs by following transcription, translation, protein synthesis [27]. The synthesized protein moves through the endoplasmic reticulum, and the Golgi apparatus gets matured and liberated back to the body [28]. Mainly, lungs get infected by the COVID-19 virus with a dry cough, fever, body pain, dyspnea-like symptoms. The virus can infect a variety of vascular functions by penetrating the respiratory tract’s epithelial cell layer and entering the circulation [29]. Consequently, the alveolar damage, hyaline membrane formation, desquamation of pneumocytes, and cellular fibromyxoid transudes can be observed in histopathology studies of an infected patient’s lungs [30].

## 4. Current Therapeutic Strategies against SARS-CoV-2

COVID-19 has yet to receive a clinically recognized therapy. So far, the therapeutic options currently used have been aimed at minimizing and managing symptoms. A few antiviral medicines are now being tested in clinical trials. These medications may operate as immunomodulators or as antiviral agents directly. Since the SARS-CoV-2 outbreak, this antiviral drug research has continued. COVID-19 control treatments are primarily focused on the disease’s pathogenesis. If targeting is done in essential viral proteins to upgrade virus internal structure and reproduction, medicines may be classified as virus targeting antivirals (VTA). If the target is a protein located inside the sick host, they may target antivirals (HTA) [31]. The pharmacological treatment for COVID-19 is currently through multiple phases of clinical studies to ensure its efficacy and safety. COVID-19 has yet to be targeted by specific antiviral medication, according to current knowledge. As a result, we must rely on our own safety rather than rely on treatment. Clinical trials should be conducted appropriately to avoid antiviral drug side effects and unpleasant responses. To avoid side effects, each patient’s condition should be considered whether antivirals are given alone or in combination with other medications. However, because drug development is an expensive and time-consuming procedure, the current COVID-19 situation necessitates the use of freshly found and more effective medicinal replacements. As a result, nanotechnology might be seen as a powerful tool in the fight against COVID-19, as it offers up new diagnostic and therapeutic possibilities [32]. The availability of the current treatment options for SARS-CoV-2 are depicted in the Table 1.

## 5. Ongoing Diagnostic Techniques for SARS-CoV-2

**Figure 2:**
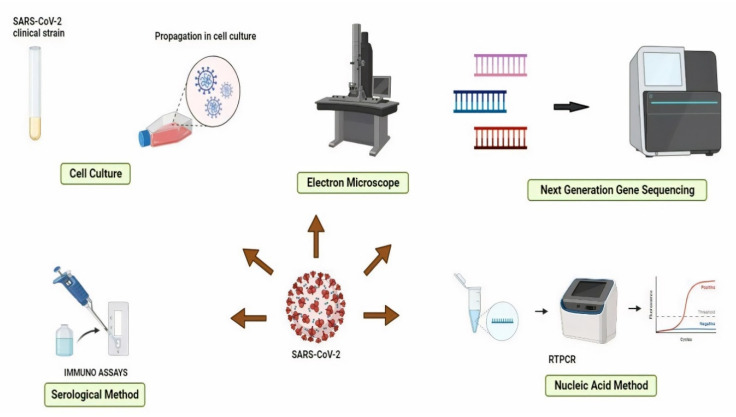
Methods for detecting RNA viruses.

To date, various standard diagnostic methods have been used to detect SARS-CoV-2 infection. RT-PCR has long been considered the gold standard for detecting RNA viruses. This methodology was developed by the Centers for Disease Control and Prevention (CDC) in the United States. The RT-PCR test examined sputum, bronchoalveolar lavage, nasopharyngeal swab, pharyngeal swab, and saliva samples [46]. The electron microscope, cell culture, serological methods, (nucleic acid methods, and next-generation sequencing methods are the five types of RNA virus screening methodologies (Figure 2). The image of the virus captured under an electron microscope (EM) is being utilized to launch ground-breaking disease detection efforts. Immunoelectron microscopy (IEM), which detects a specific antibody-antigen combination, and solid-phase IEM (SPIEM), which identifies viral particles directly on the solid surface of a matrix, are both utilized in the diagnosis of RNA viruses [47]. EM, on the other hand, has some disadvantages, including high costs, the need for well-trained workers, and limited sensitivity [48]. Cell culture, a standard process, is employed as a validation standard for most viral diagnostics procedures that have been developed. On the other hand, cell culture is unsuitable for urgent situations due to its lack of specificity and lengthy incubation period [49]. Serology-based procedures and molecular approaches are the most often employed methodology for SARSCoV-2 diagnosis. The clinical samples are withdrawn from the respiratory secretions, body tissues, or blood for investigation of the genetic material of viruses. Further, advanced technology like gene sequencing has become a commonly utilized tool, especially in the epidemiology and characterization of viruses. This method is precise and trustworthy, but its higher cost make practical usefulness limited [50].

### 5.1. Point-of-Care Diagnostics

In comparison to RT-PCR, point-of-care is a revolutionary diagnostic procedure that requires relatively little time. It assists physicians and medical workers develop suitable quarantine procedures for positive patients, allowing them to receive urgent medical attention and prevent the disease from spreading further. Point-of-Care techniques are classified as either serological antigen or molecular detection methods, with the former detecting COVID-19 in blood via lateral flow immunoassay and colloidal gold immunochromatography, and the latter detecting COVID-19 in nasopharyngeal swab, throat swab, saliva, and nasal swab via PCR technique. WHO (World Health Organization) now recommends using these new point-of-care immunodiagnostic tests solely in research settings, based on current evidence. They should not be utilized in any other situation, including clinical decision-making, unless there is sufficient evidence to justify their use for specific reasons [46].

### 5.2. Lateral Flow Immunoassay

The FDA has approved the use of a lateral flow immunoassay-based point-of-care test to diagnose COVID-19 (EUA). One test, developed by Cellex Inc., is able to detect COVID-19 IgM/IgG antibodies to SARS-CoV-2. This method can be processed in very short time and used for the qualitative identification and discrimination of IgM and IgG antibodies of SARS-CoV-2 [51]. The other developed method is Chembio Diagnostic’s DPP COVID-19 IgM/IgG, which provides results in about 15 min utilizing a simple finger–stick method [51].

### 5.3. Immunochromatography Assay by Colloidal Gold Method

This is a quick and straightforward detection approach that is commonly utilized for illness diagnosis [29,30]. With this technology, Aytu Bioscience established most reliable SARS-CoV-2 IgG/IgM Rapid Test, which can be confirmed within 10 min. Furthermore, it has been recorded higher sensitivity and specificity of IgM compared to other tests [51].

### 5.4. Signal Amplification Techniques

In a disease state, a nucleic acid-containing pathogen in samples is present in few numbers so amplification is needed to detect pathogens [52,53]. The amplification techniques are worked on the basis of following pattern:

(a) amplification of target nucleic acid,
(b) amplification of probes that interacts with the target nucleic acid,
(c) amplification of signals obtained from target nucleic acid [54].

The amplification techniques are designed into four different groups:

(a) branched DNA technique,
(b) tyramide signal amplification,
(c) hybrid capture,
(d) cleavage-based signal amplification [55].

The principle involved in the branched DNA technique is detecting a signal from target nucleic acid after immobilization and then amplification with the present multiple branched and labeled probes. In case of tyramide signal amplification, the hybridization of target nucleic acid and a biotinylated probe take place. It further promotes the complex of the formation of nucleic acid- biotinylated probe. Streptavidin containing hydrogen peroxidase is incorporated into the medium which binds to nucleic acid-biotinylated probe complex. The addition of inactivated tyramide as a substrate resulted in the formation of activated tyramide and precipitation takes place, precipitate amount indicates signal amplification [56]. The hybrid capture technique favors the hybridization of the complementary probe of signal–stranded target DNA. As a result, the transfer of the DNA: RNA hybrid initiated and transferred to the polyclonal anti-DNA: RNA hybrid antibody. The conjugated enzyme-labeled monoclonal antibody is used to label this complex. Finally, once the enzyme attaches to the chemiluminescence substrate, the signal is generated [57].

### 5.5. Molecular Methods

Molecular methods for detecting SARS-CoV-2 are mainly based on the proteomic and genomic composition of the virus. Two types of nucleic acid composition are currently available *viz* RT-PCR and RT-LAMP (reverse transcription loop-mediated isothermal amplification) [58]. Three main genomic targets are- (i) envelope protein gene (the E gene), (ii) RNA-dependent RNA polymerase gene (the RdRP gene), and (iii) nucleocapsid protein gene (the N gene) [52].

### 5.6. Loop-Based Isothermal Amplification

The loop-based isothermal amplification was created to obtain a test result that was quick, accurate, and cost-effective. Instead of the thermal cycle used in RT-PCR, amplification of the target site occurs without changing the temperature reaction [59]. The advantage of loop-mediated isothermal amplification is that the amount of DNA generated in RT-PCR is substantially higher, allowing for visualization without any additional processing. According to the study, when compared to RT-PCR, which is less sensitive, loop-based isothermal amplification has a sensitivity of more than 97% to the target open reading frame (ORF 1ab) gene. Another study found that loop-mediated isothermal amplification is precise, owing to the use of six to eight primers to determine the eight various sections of the target DNA [59,60].

### 5.7. Immunological Assays

The molecular methods are effective and sensitive for the diagnosis of COVID-19. However, it has some limitations like sampling failures, complex protocols, and expensive equipment, and these methods may sometimes give false negative results. Immunological assays are preferred to mitigate these limitations, while this technique requires comparatively more straightforward sampling with fewer technical experts and inexpensive equipment. Besides this, immunological assays are also used for antigen detection as an alternative for RT-PCR [61]. Immunological assays fall into various types, but the most common comprise antibody or antigens immobilized on a matrix that interacts with viral targets or antibodies in clinical samples. Then it is time to diagnose a virus-specific immunological response by adding more receptor protein to validate the antigen or antibody [61].

### 5.8. Antibody-Based Assays

After the virus infection has subsided, the body develops an immune response by manufacturing specific antibodies in reaction to the harmful organism. Antibody-based assays help diagnose this immune response, as well as detecting the earlier SARS-CoV-2 infection. Since the body takes time to elicit a reaction, antibody tests are rarely used to detect acute infections in their early stages because the body has not yet fully adapted to the associated antigen, which will require time to detect by establishing an immune response. As a result, antibody testing will yield false-negative findings at that time due to the lack of antibodies despite the presence of disease. Due to their short-lived nature, IgM antibodies were the first antibodies generated after a virus attack, and their identification necessitates a likely active or recent infection. Aside from that, IgG is the primary antibody that gives long-term protection against re-infection with the same virus [52] As a result, both IgG and IgM detection can provide information on the virus infection time course.

### 5.9. Antigen-Based Assay

Early and precise diagnosis of the disease and adequate quarantine conditions for both symptomatic and asymptomatic individuals are key factors in limiting the COVID-19 pandemic [62]. Since antibody-based detection assays have various limitations, such as when early detection is necessary, antibody detection will be impossible because antibodies will not form in the early stages of the disease. In order to detect viral RNA or viral antigens using antigen-based immunoassays, viral RNA or viral antigens are required. For simplicity of use, antibody-based assays should be accompanied by one of these techniques, and antigen tests that take less time are desired. Antibodies specific to viral antigens are added to the tests, which identify viral immunogen as a precursor to infection. When compared to other approaches, these are pretty quick and cost-effective. Antigen diagnosis tests may be employed for early detection of COVID-19 but not for prior exposure if the sensitivity and specificity are proven [62].

## 6. Approaches Based on Nanotechnology for Diagnosing SARS-CoV-2

At present, nanotechnology has gained much attention for detecting the novel coronavirus disease due to some known problems with the current diagnostic techniques available [63]. The current diagnostic techniques include RT-PCR (Reverse Transcriptase—Polymerase Chain Reaction), which is utilized to detect asymptomatic patients, but it was found that it also shows optimistic findings in the existence of SARS-CoV-2 to the samples. Another problem associated with this is the lack of PCR infrastructure for proving a sufficient number of kits for diagnosis in remote areas. Thus, the demand was not fulfilled [64,65]. WHO, in its report, mention the urgent need for diagnostic kits for detection of SARS-CoV-2. There are various nanotechnological methods available that can help to improve and meet the current high-level demand in diagnosis [9,66].

### 6.1. Nucleic Acid Testing

Instrumental RT-PCR diagnostic approaches are currently limited due to procedures that use isothermal conditions for nucleic acid amplification. Loop-mediated isothermal amplification is one of them, and it is thought to be a reasonably sensitive technique for detection [67,68].

Chen et al. investigated magnetic nanoparticles by using RT-PCR procedures to isolate SARS-CoV-2 viral RNA. For collecting RNA on its surface with carboxyl groups, these nanoparticles use poly(amino ester). For RT-PCR diagnosis, the carboxyl group is used in a complex of RNA magnetic nanoparticles. This procedure takes very little time and poses very little danger. Magnetic nanoparticles, rather than extraction, were utilized to isolate viral particles in another investigation. Because of the functionalized target receptors, the virus particles get sturdily linked to the nanoparticles, according to the findings. SPIONs (Superparamagnetic iron oxide nanoparticle) were used for this, as their external magnet aids in the isolation of virus particles. The other techniques such as quantitative Reverse transcription—polymerase chain reaction (qRT—PCR) assays, cell triggering assays, and immunochromatographic strip tests can be used to diagnose the virus [69]. Roh et al. combined quantum dots with fluorescent nanoparticles to develop a fast and sensitive diagnostic method. When the RNA aptamer is coupled with fluorescent quantum dots, it exhibits a difference in the optical signals of the quantum dots if the viral detection is positive. It is feasible to identify viral particle concentrations as low as 1 pmol μL^−1^ with this approach [70].

### 6.2. Point-of-Care Testing

**Figure 3:**
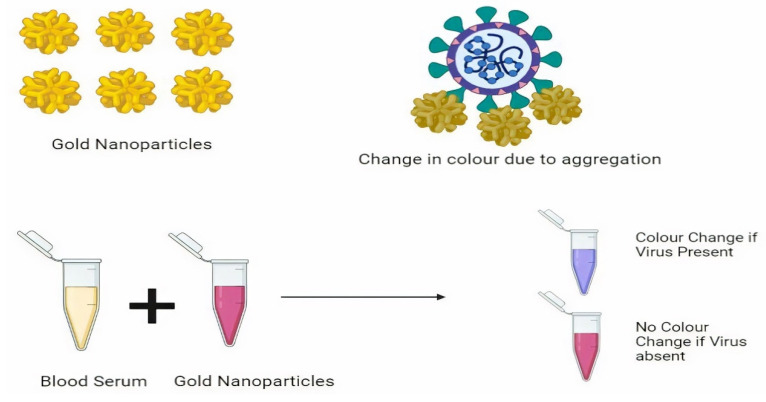
Nanoparticle-based virus detection using calorimetric assay.

Because it is a quick testing approach that eliminates the need to transfer samples to a laboratory, point-of-care testing is commonly employed in remote regions with limited facilities and infrastructure. The biosensors utilized are known as colorimetric biosensors because they detect color changes [71,72]. According to one study, paper-based DNA colorimetric sensors can detect virus samples relatively quickly. The cationic pyrrolidinyl peptide nucleic acid (PNA) probe employed in this procedure is more stable than DNA and RNA probes and is used to diagnose COVID-19 and MERS-CoV, both of which have lysine in their probe. Lysine, which is a cation, interacts with negatively charged silver nanoparticles and DNA. PNA-based nanoparticles aggregate with silver nanoparticles in the absence of viral DNA, but instead form complexes with viral particles in the presence of viral DNA. The result is based on the sample’s color change (presence or absence situations) and is determined by a paper-based analytical device (PAD) (Figure 3). It is possible to detect up to 1.53 nM with point-of-care testing [73]. Teegam et al. used gold nanoparticles to diagnose COVID-19 and MERS-CoV virus infections. Thiol groups operate as probes in their research, interacting with the upstream E protein gene and capped gold nanoparticles. In the absence of virus, nanoparticles aggregate by changing color; however, in the presence of virus, the virus forms a compound with viral DNA, blocking the aggregation process and limiting the change of optical characteristics of nanoparticles. Localized surface plasmon resonance shifts were used to diagnose the color variations. With a detection limit of 1 pmo/l.74, the method takes less time and is more cost-effective. The gold nanoparticles generate a solution that collects the virus, and the color change is used to detect it. It is a COVID-19 fast test. The main benefit of this detection method is that gold nanoparticles show specific colors by absorbing a specific wavelength. If the sample contains SARS-CoV-2, this results in a shift in the absorption peak, which causes color changes that can be seen with the naked eye when the virus concentration is high. When compared to conventional PCR procedures that need the extraction of RNA and amplification, these are a time-consuming process, and optical qualities are required for detection in point-of-care testing, hence gold nanoparticle-based techniques are chosen over other diagnostic approaches [74].

### 6.3. Biosensors Based on Electrochemistry

Biosensors based on electrochemistry are thought to be one of the utmost effective tools for detecting the COVID-19 virus. A modified electrochemical sensing technique with gold nanoparticles maintaining the biomolecule-based functional moiety was used in this approach [75]. Layqah et al. developed an immunosensor using carbon electrodes covered with gold nanoparticles. In this technique COVID-19 spike protein interacts with the virus and the antibody present in a small proportion of the sample, and changes in the current are used to detect the virus. When there is no virus in the sample, the antibody attaches to the SI protein and creates the peak current; however, when the virus is present, the antibody binds to the virus and creates a stronger peak current. The present change is measured, and a conclusion is reached. The detection limit is between 0.4 and 1.0 pg/mL [76]. For the diagnosis of SARS and COVID-19 virus, Ishikawa et al. employed nanowires in the study rather than nanoparticles. Nucleocapsid proteins were detected using In_2_O_3_ nanowire sensors and fibronectin-based antibodies. In comparison to antibodies and aptamers, antibodies resemble proteins with a higher binding capacity. The virus is detected by changes in the electric current, which are noticed as a signal [77].

### 6.4. Chiral Biosensors

It has been reported that Chiral biosensors are utmost valuable technologies for diagnosing SARS and COVID-19 virus. According to a recent report, the COVID-19 virus was diagnosed using opened chiral zirconium quantum dots. In this concern the Zirconium quantum dots and magnetic nanoparticles interact with the virus in this manner, resulting in a magnetoplasmonic glow where the virus is present. The magneto plasmonic-fluorescent nanohybrids were then isolated using external magnets, and the virus was detected by measuring the fluorescence intensity. It has a 79.15 EID/50 L detection limit [78]. A chiral immunosensor with self-assembled layers of quantum dots and chiroplasmic gold nanoparticles was used in another study. COVID-19 may be detected in blood samples by mixing gold nanoparticles and quantum dots with the viral sample; chiral optical response changes are identified using the circular dichroism method, which has a low detection limit of 47.91 EID/50 L for coronavirus [79].

## 7. Nanotechnology-Based Approaches in the Treatment of SARS-CoV-2

**Figure 4:**
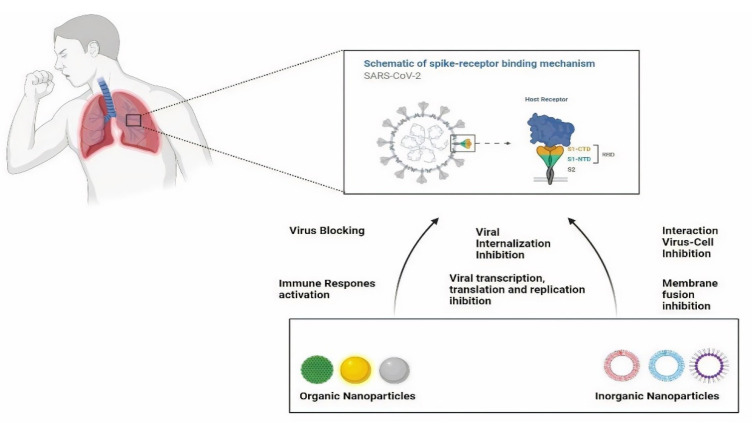
Types of nanoparticles for SARS-CoV-2 treatment.

For COVID-19 treatment, nanotechnology-based treatment has applications in membrane fusion, inhibition of virus-cell interaction, transcription, translation, viral replication, cell internalization, and activating such mechanism that causes irreversible damage to viruses. Nanotechnology-based treatment has drawn attention because of its properties. Some of the nanoparticles are discussed below. Figure 4 explores the future use of nanostructured materials to deliver recommended and repurposed antiviral medicines, which can improve treatment efficacy by reducing toxicity and allowing for controlled release.

### 7.1. Inorganic Nanoparticles

#### 7.1.1. Silver Nanoparticles

The respiratory chain can be harmed by silver nanoparticles (AgNPs), and the electron chain enzymes particularly useful in combating several viruses [80]. AgNPs’ greatest strength is that they can be used as antiviral medications with only little alterations. The researches documented that exposed AgNPs with a diameter of 55 nm have been found to have inhibitory effects for the Monkeypox Virus (MPV) and preventing virus internalization [81]. However, some restrictions of these bare AgNPs can be controlled by using polyvinylpyrrolidone (PVP), polyvinyl alcohol (PVA), etc. as coating materials.

#### 7.1.2. Gold Nanoparticles

In in vivo and in vitro investigations, AuNPs with long linkages of sulfonate undecanesulfonic acid (MUS) and mercaptoethanesulfonic acid (MES) demonstrated irreversible deformation in various viruses, including respiratory syncytial virus. AuNPs combined with MUS, which is present as a multivalent binding with the virus, are exposed in a mechanistic interaction. It also causes the capsid’s structure to collapse. As a result, this multivalent binding provides an engrossing course of action for COVID-19 therapy. AuNP was also employed against an RNA virus in one investigation. Another study discovered that the nanoparticle technology reduced membrane fusion caused by MERS-CoV. It also demonstrates the possibility of curing COVID-19 [82].

#### 7.1.3. Nanoparticles of Mesoporous Silica

The Mesoporous silica nanoparticles (MsNPs) have different pore sizes that enable molecules to accommodate outside and inside for co-delivery. The dual nature provides an outstanding manifesto for treating COVID-19 infections. These nanoparticles interact with the various ligands to stop the viral uptake to the host cell and the target-specific liberation of those ligands, it also prevents replication of the virus. Lee et al. documented significant dual delivery characteristics of the MsNPs and showed improved antiviral activity [83].

#### 7.1.4. Iron Oxide Nanoparticles

Antiviral properties of iron oxide nanoparticles (IONPs) are particularly depending on the alteration of the surface with sulfonates or other compounds. Bromberg et al. formulated core-shell silica magnetic nanoparticles containing poly (hexamethylene biguanide) functionalization (PHMBG) for improved antiviral activity. On different strains of the virus, NPs had a differential virucidal effect. It happened as a result of the genetic composition’s amino alkylation. It also prevents gene replication, resulting in viral deactivation. Because many cases of COVID-19 have demonstrated iron dysregulation [84], IONPs need to be given more consideration. Recent research suggests that SARS-CoV-2 interacts with hemoglobin (and other receptors) in erythrocyte or red blood cell progenitors, causing hemoglobin denaturation and iron metabolism dysregulation. A key concern is an excessive iron burden in the tissue [85].

### 7.2. Organic Nanoparticles

#### Carbon Nanoparticles and Graphene Nanoparticles

Carbon-based nanoparticles (CNPs), such as graphenes, carbon nanotubes (CNTs), and fullerenes nanoparticles, have intriguing physicochemical features that can be used for innovative industrial and scientific applications [86]. When it comes to COVID-19 treatment, these CNTs have some toxicity limits. The activation of macrophages in the lungs leads to fibrosis and collagen accumulation in the lesions when exposed to the lower respiratory tract [87]. Intranasal administration of CNTs causes an amplification of influenza H1N1 virus infection in lung epithelial cells. When biomolecules (polymers and proteins) were functionalized on the surface of CNTs, it was found that the inflammatory process was significantly reduced, resulting in reduced cytotoxicity in the respiratory system [88]. CNTs conjugated with hyaluronic acid increase interactions with bronchus cells, and treatment reduces inflammation in the lungs. Furthermore, CNTs and graphene quantum dots functionalized with antiviral medicines (CHI360, CHI415, CHI499, and CDF119) inhibited reverse transcriptase enzyme activity in HIV-positive patients’ cells [88]. These CNPs can be used to administer COVID 19 in the future.

### 7.3. Dendrimers

Dendrimers represent a significant advancement in nanotechnology since they can improve the efficacy of bioactive substances and medications. Dendrimers are synthetic nanoarchitects that are well-defined and highly branching [89]. It has unique physicochemical qualities such as efficient drug encapsulation, solubility, biodegradability, low polydispersity, and biocompatibility. The antiviral activity of dendrimers is amplified as a result of their strong contacts with viruses. HSV2 (Herpes simplex virus type 2) and HIV (human immunodeficiency virus) have both been treated with dendrimers, which prevent viral entrance fusion. Dendrimers made of polyamidoamine (PAMAM) are effective at preventing influenza in mice [90]. SPL7013 Gel is an example of a dendrimer available on the market (Viva Gel). It is a dendrimer with a divalent benzhydryl amine (BHA) core that works as a microbicide. The BHA core comprises four generations of lysine that bifurcate with an outermost branch, with 32 naphthalene di-sulfonic acid groups capped in the outermost branch, and it was designed to inhibit HSV and HIV infections. SPL7013 Gel’s goal is to be utilized as a superficial vaginal microbicide that has been identified [91]. Dendrimers have not yet been utilized to combat SARS-CoV-2. It can; however, be employed for the treatment of the COVID-19 infection shortly.

### 7.4. Lipid-Based Nanoparticles

Because of their excellent biocompatibility and biodegradability, lipid-based nanoparticles have a large potential to be used in COVID-19 clinical situations [92]. Many lipid-based formulations are found in commercially available drugs. AmBisome, Doxil, and other similar products are examples. Because of their amphiphilic and hydrophobic character, several forms of lipid raw materials, such as fatty acids, waxes, oils, phospholipids, mono-, di-, and triglycerides, could be employed as nanocarriers. Since their encapsulation into various classes of antivirals mediates the carriage from the administering site towards the target site while modulating distinct biological retaliation, lipid-based nanoparticles have been explored for the treatment of herpes, hepatitis C (HCV) and B (HBV), HIV viruses, and other viruses [93,94]. Liposomes are ideal nanocarriers for both hydrophilic and lipophilic compounds since they have both aqueous compartments and hydrophobic layers. Polyunsaturated liposomes had more substantial virus inhibitory potential against three viral types: HCV, HBV, and HIV, according to a study. The findings indicated that virus-associated and cellular cholesterol levels were linked to a reduction in infection. Surface-modified liposomes can boost the potential of nanocarriers. LCNPs (liquid crystalline nanoparticles) are nonlamellar self-assembled systems made up of polar lipids such glyceryl monooleate (GMO) and phytantriol (PHYT) [95]. These nanocarriers have gotten a lot of attention since they can hold imaging agents as well as tiny and large molecules in their nano compartments [96]. Nanoemulsions (NE) and microemulsions (ME) are lipid-based nanodroplets that are identical in appearance but differ in physicochemical stability. Solid lipid NPs (SLNs) and nanostructured lipid carriers (NLCs) are made up of solid and liquid lipids or solid lipids, respectively. It has been discovered that alveolar macrophages can be produced utilizing SLNs, a type of cell-related with infectious lung diseases like COVID-19 [97].

### 7.5. Polymeric Nanoparticles

Polymeric nanoparticles are used to combat virus-borne illnesses, because they can be arranged to travel to specific intracellular or extracellular targets and impede virus attachment to the host cell receptor. These features are essential to stop viruses from causing diseases and to eradicate their negative consequences [98]. The use of nanoparticles to convey active compounds has been shown to be successful in preventing virus reproduction [99]. In in vivo and in vitro models, intravenous injection of poly(lactic-co- glycolic acid), PLGA (Poly lactic-co-glycolic acid) nanocarriers improved the anti-influenza applicability of dyphylline in H1N1 infection, resulting in sustained drug release in the lungs and efficient protection against noxious dosages of the virus. The optimized NPs PEGylation is a safe formulation as it prevents activation and clearance of classical macrophages from the lungs for at least one month [100,101]. It has been proposed that replacing mAbs (Monoclonal antibodies) and compounds can reduce virus uptake in host cells. As a result, NPs can be utilized to prevent infection in humans by blocking the interaction between COVID-19 and ACE2. As a result, NPs can inhibit COVID infections by preventing the virus’s ACE2 binding. Compared to inorganic NPs, polymer-based nanoparticles or polymeric structures are subjected to pulmonary systemic absorptions and have lower cytotoxic effects in delicate cells [102]. Aerosol administration of corticosteroids-loaded polymeric NPs is utilized to treat asthma and chronic obstructive pulmonary disease with deep discernment in the lungs [102]. Because of its biocompatibility, polymeric NPs can be employed in inhalation therapy. The infected cells cause the activated immune cells in COVID-19 patients to produce a lot of inflammatory cytokines [103,104]. White blood cells engage in intense phagocytosis, resulting in the nebulization of PLGA nanoparticles overlaid with chitosan, which stops neutrophils and eosinophils from migrating to the lungs and prevents severe physiologic dysfunction [105]. Multifunctional polymeric nanocarriers have been certified as a new nano-sized stage for clinical solicitation, and they may be quickly updated for safe use in the recent SARS-CoV-2 epidemic [106].

## 8. Nano-Based Sanitizer and Disinfectants

Since the breakout of the new coronavirus, hand hygiene has been an essential tool in the fight against it. While the virus is transferred directly by coughing and sneezing, a study indicated that poor hand hygiene is responsible for 42% of infections. Hand hygiene is especially crucial in hospitals, where 40% of nosocomial infections are caused by poor hand hygiene [107]. As a result, especially in the current pandemic situation, an effective tool for maintaining adequate hand hygiene is essential. Hand sanitizers typically contain 70% alcohol, as well as other chemical or natural components. For many years, it has been the dominant force in this sector. Manufacturers are becoming more interested in nano-based sanitizers as nanoscience advances. As a result, silver nanoparticles have emerged as a viable solution for a wide range of COVID-19-related issues, including detection and disinfection [108]. Silver-based nanoparticles were found to have a wide range of bactericidal and antiviral activities in trials conducted worldwide. By inhibiting surface glycoproteins, it effectively inhibits viral RNA and reduces virulence [109]. Another benefit of nano-based hand sanitizers is that they can be made in a non-alcoholic formulation for customers who dislike alcohol-based hand sanitizers for various reasons [110]. Disinfecting the air, surfaces, and personal appliances is another strategy for neutralizing this virus [111]. While chemical disinfectants (such as chlorines, peroxides, quaternary amines, and alcohols) have been used for surface and personal equipment disinfection and sterilization, they are commonly associated with limitations such as high concentration requirements for viral inhibition and potential risk to public health and the environment [112,113]. As a result, metallic nanoparticles (silver and titanium dioxide) have been proposed as alternatives because of their broad range of applications, environmental and public safety, and efficacy at low concentrations [114]. Preliminary studies, for example, demonstrated that a coating of silver nanoclusters and silica composite on face masks had virus inhibition properties against SARS-CoV-2 [115]. In another instance, the Italian company NanoTechSurface developed a self-sterilizing solution for disinfecting surfaces using titanium dioxide and silver ions [116].

Similarly, a company named FN Nano Inc. in the United States has developed a titanium-based photo-catalytic coating that, when exposed to light, can destroy viruses by disrupting the viral membrane [116]. Nanomaterial has tremendous possibility as disinfectants owing to their unique properties. It includes intrinsic antiviral properties such as the production of reactive oxygen species to destroy viruses as well as photodynamic and photo-thermal properties. Biodegradable nanomaterials can also help to mitigate the detrimental impacts of metallic nanomaterials on human health and the environment.

## 9. Nano-Based Vaccine Development

Vaccines are biological substances that stimulate the body’s immune system to produce antibodies and memory cells to resist foreign antigen particles. Immune cells generate a cascade that kills microorganisms with identical antigens, protecting the body from infection in the future. Nanoparticle-based vaccinations have diverse functionalities that can be tailored to specific challenges presented by microbes by boosting cellular immunity [17,82,117].

### 9.1. Sub-Unit Vaccines

Sub-unit vaccinations are composed of structural components of the COVID-19 virus combined with molecular adjuvants given to boost immunogenicity by stimulating the immune system [118]. Spike protein is essential for membrane fusion and receptor binding; hence a vaccination that targets spike protein is critical. Spike protein-targeted vaccines prevent infection by pre-blocking the interaction with the ACE2 receptor, limiting the virus’s membrane fusion by the antibody [119,120,121]. Nanoparticles are created using the Novavax^®^ unique recombinant nanoparticle vaccination technology with the spike protein and identical to immunogenic viruses [122]. In Brisbane, Australia, the University of Queensland is working on a novel subunit vaccination using “molecular clamp” technology. This vaccine works by pre-blocking viral protein binding [123]. The nanoparticle-based sub-unit virus is actively proceeding [124].

### 9.2. Nucleic Acid Vaccines

The antigen-antibody reaction occurs after the virus has triggered infection. After infection, the antigen encoded by the several unique nucleic acids is expressed in the host cell. The nucleic acid vaccine, a very effective vaccination technique, was developed based on the stated principle. To elicit an effective immune response, the vaccine employs chemically generated nucleic acid. Messenger ribonucleic acid (mRNA) vaccines have improved immunogenic characteristics because they can replicate the infectious process. The effects are maximized by mixing different mRNA in a single vaccination [125,126]. The use of nanotechnology in nucleic acid vaccines aids in vaccine delivery effectiveness. The introduction of nanotechnology, nanoparticles, such as cationic liposomes, polymeric nanoparticles, or dendrimer nanoparticles, can improve the stability and delivery efficacy of nucleic acid-based vaccines [127,128].

### 9.3. NP-Based Vaccines

Several investigations have shown that nanotechnology is being used to improve vaccine efficacy. In recent studies, virus-like particles are appropriate for vaccine and treatment procedure development. Nano-sized virus-like particles having viral character can be effectively delivered through lymph and capillaries [129,130]. By activating the B cell and boosting the immune system the nano-sized virus-like particles works in the host cell, which leads to the development of vaccines of nano-sized virus-like particles [131,132,133]. Nano-sized virus-like particles effectively prevent viral effects by boosting the immune system [134,135]. Using recombinant S, membrane, and envelope proteins, the nano-sized virus-like particles can be synthesized from the tested virus [136]. Nano-sized virus-like particles have an extensive range of applications and modulate vaccine safety and usefulness and have purpose-specific advantages [137].

## 10. Non-Invasive COVID-19 Detection

COVID-19 caused a viral pandemic by impacting the global economy, health system, transportation, and all elements of human life. The capacity to detect COVID-19 infections early via a non-invasive approach can function as an epidemic tool for control. Shan et al. developed a non-invasive intelligent nanomaterial-based hybrid sensor array with multiplexed capabilities for the detection and monitoring of COVID-19-specific volatile organic compounds (VOC) mixtures from exhaled breath. The potency of the device was investigated in the case control clinical research by the healthcare agencies. The sensor of the device contains various gold nanoparticles that are coupled to organic ligands. VOC exposure causes changes in the sensing layer, which might cause the organic ligand to expand or shrink. The inclusion of inorganic nanoparticles promotes electrical conductivity production. The changes in the electrical conductivity of the device indicate the presence of COVID-19 viral agents. The stated test could assist in rapid screening of large populations due to its simplicity and high sensitivity, and it could also be utilized as a valuable technique for COVID-19 screening [68]. The investigation of COVID-19 through the VOC mixtures from exhaled breath by using sensing device, it gives effective diagnosis of COVID-19 virions in less time. COVID-19 dissemination can be efficiently controlled if the device is integrated with the face mask. To achieve this aim, a concerted effort is required, but still the global community as a whole should strive for it [138]. Miripour et al. developed an efficient electrochemical sensor to assess the level of reactive oxygen species (ROS) in a sputum sample. Over 97% of positive patients were identified, and the sensors confirmed the diagnosis in under 30 s. During this pandemic, it could be utilized as a significant companion in rapidly detecting individuals who require additional medical evaluation, but it might be employed in the future to reduce the number of cases that require a CT-Scan for COVID-19 diagnosis. COVID-19 early-stage screening could benefit from these enhanced electronic biosensors [139].

## 11. Preventive Procedure from Virus

As the spreading of the COVID-19 virus is increasing globally, various guidelines are being issued by the World Health Organization (WHO) for disease control and prevention [140,141]. It is a personal feeling that virus prevention can be done by taking some measures and incorporating them into our daily life. In the localities where there is a widespread transmission of the COVID-19 virus, potential exposures are controlled by various preventive strategies. Temporary lockdowns are imposed to bring down the public gathering. Curfews are also being incorporated. A more significant number of COVID-19 positive cases are detected than these places marked as containment zones or hot spot regions, where traveling is wholly restricted to prevent further spread of the virus. Preventive measures are a new strategy to combat the virus. Early detection and proper diagnosis are necessary to prevent further spread. Preventive measures are chiefly focused on the isolation of COVID-19 positive patients. The primary strategy to combat the virus is to frequently wash hands, use portable hand sanitizer, and maintain social distance anywhere to avoid direct contact with face and mouth.

To create awareness among the general public posters and brochures prepared by many organizations related to preventing the COVID-19 virus should be widely used in all areas. The native should be asked to maintain social distancing and should stay at home as much as possible unless it is necessary to go out. In a public gathering maintaining 2 m (6 feet) distance from other people should be practiced. Such social distance is designed to bring down public interactions in a community. Individuals are suffering from infections and are yet to be identified and isolated [142,143]. By maintaining social distancing, chances of transmission can be controlled. Social distancing includes closure of offices, schools, colleges, canceling public gatherings, market suspension, encouraging online classes, office work from home, online shopping etc. Wearing of a mask by covering the nose and mouth in public is encouraged in many countries. Mask such as N95, surgical mask, and 3 ply masks prevents transmission of the virus. Wearing a double mask is encouraged for better protection. Respiratory hygiene is maintained by covering the face while coughing and sneezing. Frequent hand washing by soap or hand wash should be practiced, using a hand sanitizer containing not less than 70% of alcohol is an alternative to handwashing where water is not available, or handwashing is not possible if our hands are not filthy.

Proper handwashing should be done. We should remove the accessories such as wristwatches, finger rings before cleaning our hands. We are followed by wetting hands with water. Then, apply soap or hand washes and rub our hands thoroughly for at least 20 s and rinsing them well, followed by drying our hands with tissue paper or a clean towel and using the same tissue to turn off the faucet. Individuals are counseled to avoid touching their face, especially the nose, mouth, and eyes. We know that cleanliness is next to godliness. Here to prevent the infection, cleaning and disinfection play a vital role. High touching areas, including door handlers, window, bedside table, and switches, should be daily disinfected with surface sanitizers or household disinfectants containing dilute beach solution. The bathroom and toilet should be cleaned and disinfected regularly using a dilute bleach solution, having one part of bleach and nine parts of water. Our daily-wear clothes and hand and bathing towels should be cleaned using laundry soap or detergent. The wearing of disposable gloves while cleaning is also advised. All these measures should be taken to avoid the spread of the virus, such as wearing of mask in public, sanitizing of hands, maintaining social distancing etc. If any symptoms arise, immediately isolate yourself, consult a physician, check for temperature twice, and take proper care. Moreover, most importantly, “do not panic”.

## 12. Future Perspectives for COVID-19 Treatment and Diagnosis

### 12.1. Theranostic Nanoparticles

The use of nanoparticles has proven to be transformative in the accurate detection and effective in treating diseases. Nanoparticles with reduced toxicity, chemical flexibility, electrical charge, and compact design serve to alleviate several issues with generic medication administration routes. COVID-19 can be treated by focusing on the entry and life cycle treatment. The virus’s entry into the host cell can be inhibited by preventing membrane fusion, facilitated by the virus spike protein. We can use therapeutic nanoparticles to pre-block the entry of the COVID-19 virus by reducing the S protein’s interaction to the host cell. Viruses such as HIV1 and HIV2 [144,145,146], hepatitis B virus [147,148,149,150], hepatitis C virus [151], influenza A and B viruses [152,153,154], Herpes simplex virus type 1 and 2 [155,156,157], EBOV [158], and human norovirus [159] can all be treated with nanotechnology, and it is already being utilized commercially. Dexamethasone is a medicine used to treat the COVID-19 virus developed utilizing nanotechnology and works as an anti-edema and anti-fibrotic [160].

### 12.2. Nanotechnology-Based Intranasal Delivery Therapy

Recent research has focused on creating innovative methods for safely and effectively delivering nanoparticles into the nasal cavity as a therapy for viral infection [161]. Because the SARS-CoV-2 virus infects the mucosal layer of the nasal cavity and eye, mucosal therapy is regarded as a viable technique in treating SARS-CoV-2. Nanoparticle delivery through the nasal canal is thought to be both simple and convenient. It is also non-invasive, and the nanoparticles are quickly absorbed due to the enormous surface area and the presence of a sizeable capillary plexus in the cavity [162]. When transporting nanoparticles into the nasal cavity, factors like form, size, and surface area play a crucial role in ensuring a safe and effective treatment [163]. Small animals are used in the studies, and nanoparticles are injected into their nasal cavities. To date, inorganic, organic, and virus-like nanoparticles have been developed. It assures that the delivery is safe and effective for therapeutic needs.

### 12.3. Treatment Using Virus-Like Nanoparticles

Capsids composed consisting of structural proteins and adjuvants derived from viruses are known as virus-like nanoparticles. The capacity of virus-like nanoparticles to generate an immunogenic motif increases immunogenicity. Virus-like nanoparticles can also act as adjuvants, and changing the adjuvants can result in a far more potent immune response than viruses [136,164,165]. Intranasal delivery of virus-like nanoparticles utilizing the influenza virus has been proven to operate as a vaccine by generating many T cells and antibodies that can trigger various types of immunological responses to increase immunity and reduce recurrence [166].

### 12.4. Treatment Using Cell-Derived Vesicles

Exosomes and cell membrane-derived nanovesicles have previously been shown to have the ability to bind and eliminate bacterial toxins [167,168,169]. On the other hand, biomimetic synthesis has recently been employed to synthesize cell membrane-derived nanovesicles containing proteins with the same structure and activity as native cells [170,171,172]. By expressing high quantities of ACE2 and a large number of cytokine receptors, cell membrane-generated nanovesicles can engage with host cells for virus and cytokine binding. In investigations, nanodecoy has been shown to successfully bind and neutralize inflammatory cytokines such GM-CSF and IL-6 and prevent SARS-CoV-2 proliferation and infection [173,174]. As a result, using cell membrane produced nanovesicles as a therapeutic alternative to SARS-CoV-2 and cytokine storms could be a realistic option.

### 12.5. Pulmonary Delivery Using Nanoparticles Inhalation Aerosols

One of the benefits of administering medicine through the nasal cavity is that it affects the nasal cavity’s mucous membrane, where the infection is most prevalent. The lungs are the next target for COVID-19 infection treatment [175,176,177]. Inhaled aerosols can thus be utilized as a non-invasive and effective route of delivery. The targeted medication delivery system in the lungs also decreases the high drug concentration in the blood induced by oral and intravenous administration methods. A variety of nanotechnologies have been employed to create NPs that could be employed as lung inhalation aerosols. Drugs are given directly to the lungs via dry powder inhalers, colloidal dispersions sprays, and pressured metered-dose inhalers [174]. The delivery of high molecular weight drugs to the lungs is being studied [178,179]. Other factors to consider when delivering high molecular weight medications to the lungs include cytotoxicity and biocompatibility.

## 13. Conclusions

Many people have died due to the COVID-19 outbreak, with casualties coming from all corners of the world. The grievous nature of this pandemic has uncovered and necessitated new strategic approaches to curbing disease transmission. Because of the low efficacy and incidence of disastrous adverse drug reactions, novel COVID-19 therapies must be developed and implemented. Working on preventative and new technologies to combat infectious pandemics, which are equally as vital as the curative approach, is crucial when dealing with pandemics. The nano-based medicines are more effective compared to the conventional dosage forms. Despite these advantages, it has certain limitation such as stability, clinical study, primarily toxicity and large-scale production, all of which provide a challenge to manufacturers. Additionally, biocompatibility, nano-biointerface, safety, and regulatory issues are among the big obstacles for the manufacturers. As a result, research must be targeted in order to overcome these complications and produce better formulations. The different groups of scientists, particularly in nanomedicine, are working on their development using bioinformatics methods. Scientists have a lot of options when it comes to regulatory research. It has the potential to gain national and international exposure. The encouraging findings can also be used to patent the works in a major way.

The diagnosis of SARS-CoV-2 infection is frequently confused as either influenza or seasonal upper respiratory system viral infections. The following trustworthy, high-sensitivity, simple, rapid, and cost-effective diagnostic tools, namely RT-PCR, point-of-care, lateral flow immunoassay, immunochromatography assay, signal amplification, immunological assays, antibody-based assays, and antigen-based assay are used for the confirmation of COVID-19 infection. Furthermore, the study proved the applicability of nanotechnology-based treatment of SARS-CoV-2 employing diverse nano carriers such as inorganic nanoparticles, organic nanoparticles, dendrimers, lipid-based nanoparticles, and polymeric nanoparticles in nano formulations of antiviral drugs. When compared to traditional dosage forms, these formulations show superior therapeutic efficacy and better pharmacokinetic attributes. Similarly, nanoparticle-based vaccines, theranostic nanoparticles, nano-based intranasal delivery therapy, virus-like nanoparticles, cell-derived vesicles, and pulmonary delivery using nanoparticles inhalation aerosols all boost therapeutic efficacy significantly. While nanomedicine is a promising tool in the battle against pandemics, but it faces substantial challenges in clinical implementation, primarily toxicity and large-scale production. This review allows researchers to do study and develop better formulations for treating COVID-19 in society.

## Figures and Tables

**Table 1:** Summed up the current SARS-CoV-2 treatment options.

Drugs	Pharmacological Mechanism	Therapeutic Utility	Cons	Summary of Evidence	References
VIRUS TARGETING ANTIVIRAL (VTA)					
Remdesivir (Nucleotide analog)	Inhibit viral replication via interacting with RNA-dependent PNA Polymerase (RdRp)	SARS-CoV; Middle East respiratory syndrome-CoV (MERS-CoV); Influenza; and Ebola	There is no proof of safety or efficacy. Adverse effects like renal failure and increased liver enzymes	Remdesivir effectiveness was demonstrated in a study with 53 individuals infected with SARS-CoV-2, according to Gilead Sciences. The findings revealed a lower death rate in severe instances.	[33,34]
Ribavirin (Ribonucleic analog)	Interferes with DNA and RNA viral replication and suppresses natural guanosine synthesis by direct action on enzyme inosine monophosphate dehydrogenase.	SARS; MERS; Respiratory syncytial virus (RSV) infection; and Hepatitis C	Adverse effects like hematologic toxicity, high risk of anemia, and teratogenic effect in pregnant women.	Ribavirin is primarily used in combination with Ritonavir/Lopinavir and IFN-β.	[35]
Favipiravir (Viral RNA-dependent RNA polymerase inhibitor)	It prevents viral growth by inhibiting RNA polymerase activity.	Ebola; Influenza A	Efficacy is yet unknown. Adverse effects like hyperuricemia, increased liver enzymes, diarrhea, decreased neutrophil count, and teratogenicity.	According to a recent study, favipiravir suppressed COVID-19 development and favored viral clearance.	[34,36]
Ritonavir/ Lopinavir (Protease inhibitor)	Inhibition of the viral PL-pro or Mpro (3CL-pro)	HIV type 1	Adverse effects like hepatotoxicity, pancreatitis, cardiac conduction abnormalities, increase the risk of cardiac arrhythmia.	In a clinical study with 199 individuals, lopinavir did not show therapeutic improvement.	[37]
VIRUS-HOST INTERACTION					
Chloroquine and Hydroxychloroquine (Anti-malarial)	Both chloroquine and hydroxychloroquine block glycosylation of ACE2 receptor chains in SARS-CoV-2, reducing ligand binding and preventing the viral S protein from mediating cell entrance.	Chloroquine-Malaria; SARS-CoV; and extra-intestinal amoebiasis Hydroxychloroquine-Malaria; lupus erythematosus	Long-term use of chloroquine and hydroxychloroquine increasing the risk of retinal damage, hypoglycemia, and cardiac arrhythmias.	A recent investigation with 96,032 patients looked at the effectiveness of chloroquine or hydroxychloroquine, which is frequently administered in combination with a second-generation macrolide. The study found that chloroquine is linked to a greater incidence of ventricular arrhythmias.	[37,38]
Umifenovir (Fusion inhibitors)	Umifenovir prevents viral genes from entering the nucleus by impeding the interaction between ACE2/S protein and the fusion of the viral lipid envelope with the host cell membrane.	SARS-CoV-2; Influenza A and B	Common side effects like increased liver enzymes, gastrointestinal intolerance, and allergic reactions.	Umifenovir monotherapy was shown to be more effective than ritonavir/lopinavir in lowering viral load in COVID-19 patients.	[39,40]
Camostat Mesylate (Transmembrane protease, serine 2 inhibitors)	SARS-CoV-2 invasion into host cells is prevented by inhibiting TMPRSS2-mediated glycoprotein activation.	Chronic pancreatitis	Adverse effects like gastrointestinal intolerance, itching, skin rashes, and increased liver enzymes.	Camostat mesylate significantly inhibited the entrance of MERS-CoV, SARS-CoV, and SARS-CoV-2 into the lung cell line Calu-3, resulting in cytotoxic consequences. Furthermore, in cell line investigations, camostat mesylate therapy decreased SARS-CoV-2 infection.	[41]
HOST-TARGETING ANTI-VIRAL					
Convalescent plasma	Direct viral neutralization and immune system control molecules (cytokine storm, Th1/Th17 ratio, complement activation, etc.). Immunomodulation of a hypercoagulable condition.	SARS-CoV; MERS-CoV; H1N1; Spanish influenza A; and Sepsis	Expensive. The effects of passive vaccination are generally short-lived.	After receiving a convalescent plasma infusion, five patients with severe COVID-19 showed considerable clinical improvement. Using convalescent plasma therapies, 10 patients with COVID-19 were also cured in another clinical research.	[42]
Corticosteroids (Steroid hormones)	The interaction with glucocorticoid steroid (GR) receptors in the cell cytoplasm neutralizes pro-inflammatory cytokines, preventing inflammation.	Inflammation	Increase the risk of secondary infection	There is some benefit in the early stages of infection, and its effectiveness in the treatment of COVID-19 is still being debated by the World Health Organization.	[43]
Sarilumab (Recombinant humanized mAb, IL-6 antagonist)	Interaction with the IL-6 receptor inhibits IL-6 signaling.	Cytokine release syndrome; Rheumatoid arthritis	Still, there is not enough evidence to suggest sarilumab for COVID-19 therapy.	The use of sarilumab is under investigation.	[44]
Tocilizumab (Recombinant humanized mAb, IL-6 antagonist)	Interaction with the IL-6 receptor reduces inflammatory responses and protects against immunological dysregulation caused by inflammation.	Cytokine release syndrome; Rheumatoid arthritis	Expensive. Adverse effects like hepatotoxicity, gastrointestinal perforations, hypertension, and hypersensitivity reactions,	The research found that after a few doses of administration, C-reactive protein levels in 15 individuals with COVID-19 decreased, improving their medical state.	[45]
